# A Four−Gene-Based Risk Score With High Prognostic Value in Gastric Cancer

**DOI:** 10.3389/fonc.2021.584213

**Published:** 2021-09-02

**Authors:** Bingdong Zhang, Yuerui Li, Liu Yang, Yongbing Chen

**Affiliations:** ^1^Department of Gastrointestinal Surgery & Department of Clinical Nutrition, Beijing Shijitan Hospital, Capital Medical University, Beijing, China; ^2^Geriatric Cardiology Department of The Second Medical Center & National Clinical Research Center for Geriatric Diseases, Chinese People's Liberation Army of China General Hospital, Beijing, China; ^3^State Key Laboratory of Biomembrane and Membrane Biotechnology, School of Medicine, Tsinghua University, Beijing, China

**Keywords:** gastric cancer, risk score, mutations, prognostic value, genome

## Abstract

**Background:**

Gastric adenocarcinoma is an important contributor to cancer mortality and morbidity. This study aimed to explore the prognostic value of mutation patterns in gastric adenocarcinoma.

**Materials and Methods:**

We extracted somatic mutation data for 437 gastric adenocarcinoma samples from The Cancer Genome Atlas (TCGA) Stomach Adenocarcinoma (STAD) cohort. Kaplan–Meier survival in the R package maftools was used to analyze associations between mutations and survival. Multivariate Cox proportional model was used to establish risk formula. A four-gene-based risk score was developed to predict the overall survival of patients with gastric adenocarcinoma. We used the Tianjin cohort dataset with survival information to further evaluate the clinical value of this mutation signature.

**Results:**

Forty-five survival-related mutated genes were identified and verified, most of which were co-occurring in their mutation pattern and co-occurring with MLH3 and polymerase ϵ (POLE) mutations. Gastric adenocarcinoma samples with the 45 mutated genes had a significantly higher mutation count. Four-gene [UTRN, MUC16, coiled-coil domain-containing protein 178 (CCDC178), and HYDIN] mutation status was used to build a prognostic risk score that could be translated into the clinical setting. The association between the four-gene-based signature and overall survival remained statistically significant after controlling for age, sex, TNM stage, and POLE mutation status in the multivariate model [hazard ratio (HR), 1.88; 95% CI, 1.33–2.7; p < 0.001]. The prognostic significance of the four-gene-based risk score identified in TCGA cohort was validated in the Tianjin cohort.

**Conclusion:**

A four-mutated gene risk formula was developed that correlated with the overall survival of patients with gastric adenocarcinoma using a multivariable Cox regression model. In two independent genomic datasets from TCGA and Tianjin cohorts, low risk scores were associated with higher tumor mutation loads and improved outcome in patients with gastric adenocarcinoma. This finding may have implications for prognostic prediction and therapeutic guidance for gastric adenocarcinoma.

## Introduction

Gastric adenocarcinoma is an important contributor to cancer mortality and morbidity, and its molecular mechanism remains largely incomprehensible. Next-generation sequencing (NGS) technology could provide genomic-level information about the mechanism of cancer. Numbers of large-scale genomic analyses on gastric adenocarcinoma have been completed, including The Cancer Genome Atlas (TCGA) project.

Recent research has shown that gastric adenocarcinoma is a heterogeneous disease. Surgical resection is still the main means of curative treatment for gastric adenocarcinoma. However, a portion of patients with advanced gastric adenocarcinoma developed local recurrences and distant metastases and had a poor prognosis after resection (1). Patients would have received the best treatment if their prognosis was depicted in advance. However, different prognoses of patients with similar clinical stages or pathologic grades remain unpredictable (2–5). Profiling the genetic mutation of gastric adenocarcinoma that influences the prognosis and accurate risk assessment based on genetic screening will lead to more effective clinical strategies in precision medicine.

In this study, we identified and verified 45 survival-related mutated genes with bioinformatics analysis from TCGA Stomach Adenocarcinoma (STAD) cohort. We investigated the function of these genes *via* Gene Ontology (GO) analysis. Through random survival forest algorithm, we ranked these mutated genes by importance and constructed a four−gene-based risk score with multivariable Cox regression model. Using the Tianjin cohort dataset with survival information, we evaluated the clinical value of the risk score.

## Materials and Methods

### Stomach Adenocarcinoma Datasets

Genomic data of gastric adenocarcinoma somatic mutation and gene expression data for 437 gastric adenocarcinoma samples in TCGA data portal (level 3) were downloaded from Genomic Data Commons (https://portal.gdc.cancer.gov) (Release Date: August 23, 2018). The Tianjin cohort contained data from 78 patients from northern China (6). Frozen tissue samples derived from surgical resection specimens of primary gastric adenocarcinoma from 294 northern Chinese patients without preoperative chemotherapy or radiotherapy were obtained from the Tianjin Medical University Cancer Institute and Hospital-National Foundation for Cancer Research Joint Tissue Banking Facility. Whole-exome sequencing was performed on 78 samples. Germline DNA was obtained from matching blood samples and used as a reference sequence to detect somatic mutations. Histopathologic diagnoses were independently reviewed by at least two experienced pathologists. Clinical follow-up data were complete for 78 participants with 25.08 months of median follow-up (32 deceased, 41.03%) (**Supplementary Table 1**). TCGA cohort also has the follow-up and vital status of patients (**Supplementary Table 2**). This study was approved by the Chinese PLA General Hospital (Beijing), which waived additional informed consent because all data used in this study were obtained from public databases. This study met the publication guidelines provided by TCGA (http://cancergenome.nih.gov/publications/publicationguidelines). All data were processed and analyzed by Excel 2010 and R (version 3.5.0).

### Prognosis

Kaplan–Meier survival analyses implemented in the R package maftools and survival were used to analyze the correlation between mutations and survival (7). The log-rank test was used to determine significant differences of survival curves stratified by mutations. A two-sided p < 0.05 was considered statistically significant. Correlation between mutations and survival was also explored by multivariate Cox regression analyses by the R package survival.

### Gene Ontology Pathway Analysis

The GO pathway analysis mutated genes were annotated by the R package of clusterProfiler (8). The cutoff p.adjust value was 0.01.

### Random Survival Forest Algorithm

Random survival forest algorithm implemented in the R randomForestSRC was used to rank the survival-related genes by their importance.

## Results

### Survival in The Cancer Genome Atlas Stomach Adenocarcinoma Cohort

**Figure 1:**
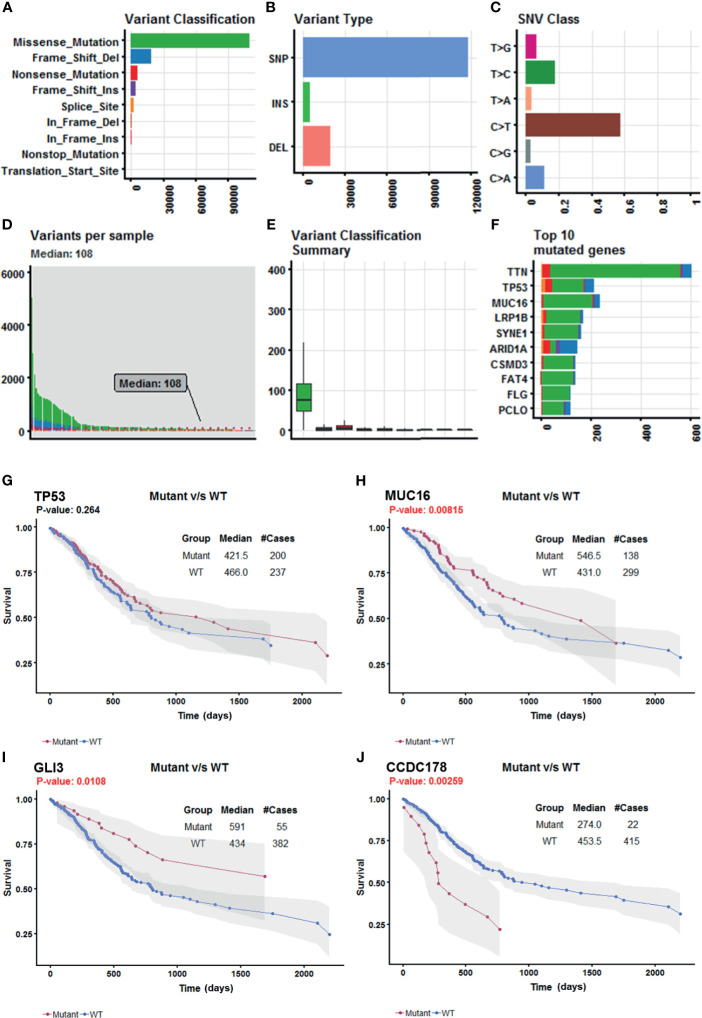
Selection of survival-related mutated genes in The Cancer Genome Atlas (TCGA) Stomach Adenocarcinoma (STAD) cohort. **(A–C)** Characteristics of the variations in TCGA STAD cohort. Histogram summarizing the variant types of all cases with substitutions, insertions, deletions, and SNP (single nucleotide polymorphism). **(D)** Stacked bar showing the cumulative frequency of variation for individual cases. **(E)** Boxplot summarizing the number of cases for each type of variant. **(F)** Stacked bar showing top 10 mutated genes. **(G–J)** Kaplan–Meier survival analyses stratified by TP53, MUC16, GLI3, and coiled-coil domain-containing protein 178 (CCDC178) mutation status, respectively. This analysis was implemented in the R package maftools.

Somatic mutation data for 437 gastric adenocarcinoma samples from TCGA STAD cohort were extracted by maftools version 1.6.15 (http://www.bioconductor.org/packages/release/bioc/vignettes/maftools/inst/doc/maftools.html). These somatic mutations included point mutations and insertions/deletions (indels). We annotated 17,431 protein-coding genes with somatic mutations that mostly consist of missense mutations (**Figure 1A**), and single-nucleotide polymorphism (SNP) was more common than insertion–deletion (InDel) (**Figure 1B**). Furthermore, the mutational contexts are derived from combinations of six mutational types (i.e., T>G, T>C, T>A, C>T, C>G, and C>A) (**Figure 1C**). The somatic mutation rates varied considerably among the samples (**Figures 1D, E**), though an average of 108 mutations occurred in each sample (**Figure 1D**). The top 10 mutated genes were *TTN, TP53, MUC16, LRP1B, SYNE1, ARID1A, CSMD3, FAT4, FLG*, and *PCLO* (**Figure 1F**).

In order to select the most weighted genes associated with survival outcome, Kaplan–Meier survival in the R package maftools was used to analyze associations between mutations and survival for each of the 17,431 protein-coding genes (**Figure 1B**). A two-sided p < 0.05 was considered statistically significant. The most studied tumor repressor gene TP53 was analyzed for example. All samples were categorized into two groups representing the wild-type and mutated TP53 respectively, and Kaplan–Meier survival analysis showed that there were no significant associations between TP53 mutations and survival in TCGA STAD cohort (**Figure 1G**). Forty-four mutated genes, such as MUC16 and GLI3, occurring in more than 5% of the patients, were significantly associated with a better survival outcome (**Figures 1H, I**). Only coiled-coil domain-containing protein 178 (CCDC178) mutations were significantly associated with a poor survival outcome (**Figure 1J**). The 45 mutated genes were listed in **Table 1**.

**Table 1:** Survival-related mutated genes by Kaplan–Meier survival analyses.

Gene (Gene Symbol)	Median time in days	Median time in days	Mutated samples	Log-rank p-value
MUC16	546.5	431	138	0.00815
CSMD1	595	424	79	0.03983
GLI3	591	434	55	0.0108
PTPRT	503	436	50	0.02372
CDH23	600	429.5	48	0.00794
COL11A1	549	434	46	0.01143
MACF1	600	433.5	44	0.03886
CELSR3	679	426.5	43	0.00691
TENM3	485	439.5	43	0.02574
DCLK1	594	435	43	0.04194
TECTA	657.5	434	42	0.01237
ATM	688	428	39	0.02031
ITPR3	664	426.5	38	0.01208
ZNF462	762	422	37	0.02954
UTRN	522	439	36	0.01643
RANBP2	544	435	36	0.02873
HERC1	589.5	427	35	0.0406
FBXW7	644	434	35	0.0493
CHD6	569	434	32	0.0354
CPAMD8	669	427.5	31	0.03831
ANK1	639	434	30	0.01384
HYDIN	468	443	30	0.03202
CTNNB1	742	427.5	29	0.00461
ST8SIA6	695.5	427	29	0.04623
PCDH20	766	427.5	28	0.01026
KIF26B	640	431	28	0.03642
RAI1	670	428	28	0.04695
YLPM1	713	436	26	0.00804
PARD3B	809	431	26	0.01547
GREB1	805	432.5	26	0.02778
SOGA3	616	437.5	26	0.032
COL15A1	588	437.5	25	0.02058
ABCC8	812	427.5	24	0.02221
MYH14	668	440	24	0.0243
KIAA1549	678.5	431	24	0.04694
CDHR2	900	429.5	23	0.00268
MYH11	491	439.5	23	0.02104
PCDHB6	600	414	23	0.02661
COL5A2	900	432.5	23	0.04145
CCDC178	274	453.5	22	0.00259
ZZEF1	679	429.5	22	0.00588
THSD1	514.5	440	22	0.01973
DLGAP2	655.5	428	22	0.02265
OBSL1	616	435	22	0.03932
LAMB3	736.5	440	22	0.04177

To elucidate the function of these survival-related mutated genes, we conducted GO analysis and revealed that many genes play an important role in “cell–cell adhesion *via* plasma–membrane adhesion”, “extracellular matrix component”, and “alpha-catenin binding”, which were highly correlated to cancer metastasis and invasiveness (**Table 2**).

**Table 2:** GO analysis of 45 survival-related mutated genes (partial data).

Genes	p-value	p.adjust	Annotations
Biological process			
7	7.50E-08	0.000105	GO:0007156:homophilic cell adhesion *via* plasma membrane adhesion molecules
7	1.92E-06	0.001342	GO:0098742:cell-cell adhesion *via* plasma-membrane adhesion molecules
Molecular function			
5	5.89E-05	0.0048705	GO:0051015:actin filament binding
3	7.38E-05	0.0048705	GO:0030020:extracellular matrix structural constituent conferring tensile strength
Cellular component			
4	3.91E-06	0.000673	GO:0044420:extracellular matrix component

Partial data, genes involved p.adjust < 0.01 (GO analysis).

GO, Gene Ontology.

### Clinical Features of Patients in The Cancer Genome Atlas Stomach Adenocarcinoma Cohort With the 45 Mutated Genes

**Figure 2:**
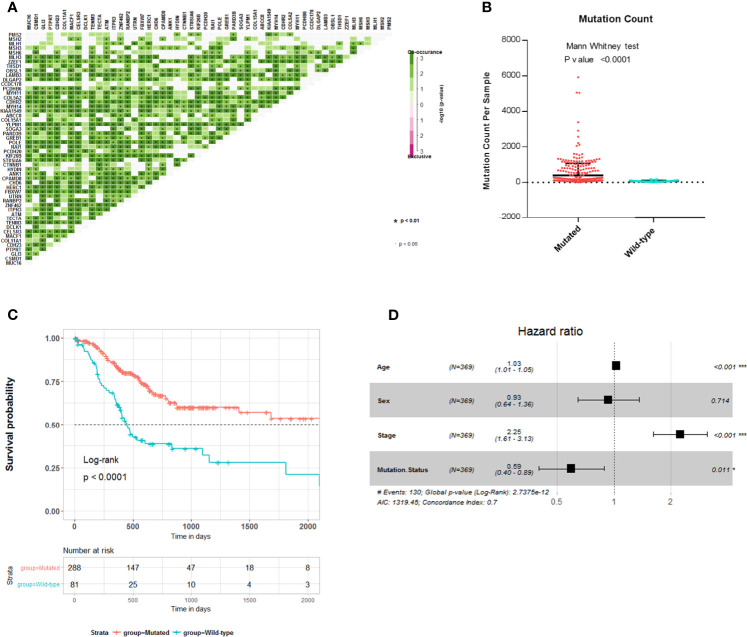
Association of 45 mutated genes status with tumor mutation load and prognosis in The Cancer Genome Atlas (TCGA) Stomach Adenocarcinoma (STAD) cohort. **(A)** Mutually exclusive and co-occurring gene pairs in STAD displayed as a triangular matrix. Green indicates tendency toward co-occurrence, whereas pink indicates tendency toward exclusiveness. **(B)** Mutation count per sample of gastric adenocarcinoma stratified by 45 mutated genes status. **(C)** Kaplan–Meier survival analysis stratified by 45 mutated genes status. **(D)** Multivariable Cox regression model tests for age, sex, TNM stage, and 45 mutated genes status. Square data markers indicate estimated HRs. Error bars represent 95% CIs. *p < 0.05; **p < 0.01; ***p < 0.001.

To explore the relationship between these survival-related mutations, we performed pair-wise Fisher’s exact test to detect significant pairs of mutated genes in the 45 genes and DNA mismatch repair (MMR)-related genes [PMS2, MSH2, MLH1, MSH3, MLH3, MSH6, polymerase ϵ (POLE)]. Interestingly, most of the 45 mutated genes were co-occurring in their mutation pattern and co-occurring with MLH3 and POLE mutations (**Figure 2A**). Tumor mutation burden (TMB) is an important determinant for molecular subtyping of gastric adenocarcinoma in TCGA (9). Recent studies have shown that gastric adenocarcinoma with POLE mutations or microsatellite instability–high (MSI-H) had DNA MMR signatures and higher TMBs (10). Gastric adenocarcinoma samples with the 45 mutated genes had a significantly higher tumor count (**Figure 2B**; Mann–Whitney test p-value <0.0001). Since these mutations tended to occur simultaneously, we explored the relationship between all these mutations and prognosis. Kaplan–Meier survival analysis showed that the 45 mutated genes were significantly associated with a better survival outcome in TCGA STAD cohort (**Figure 2C**; log-rank test, p < 0.0001). Multivariate Cox regression analyses showed that the correlation remained statistically significant after controlling for confounding factors such as sex, age, and TNM stage (**Figure 2D**). So, the 45 mutated genes were important prognostic indicators associated with tumor mutation load.

### Construction of Risk Score Formula

**Figure 3:**
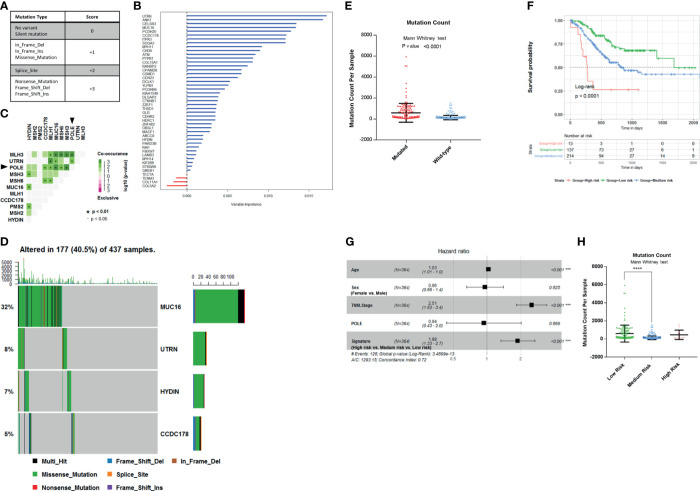
Establishment of survival-related risk score in The Cancer Genome Atlas (TCGA) Stomach Adenocarcinoma (STAD) cohort. **(A)** Mutation score matrix. **(B)** Variable importance of the 45 survival-related mutated genes. **(C)** Mutually exclusive and co-occurring gene pairs in STAD displayed as a triangular matrix. Green indicates tendency toward co-occurrence, whereas pink indicates tendency toward exclusiveness. **(D)** Mutation count per sample in non-synonymous mutations. Frequency of MUC16, UTRN, HYDIN, and coiled-coil domain-containing protein 178 (CCDC178) mutations and gene mutation patterns. **(E)** Mutation count per sample of gastric adenocarcinoma stratified by four-gene mutation status. **(F)** Kaplan–Meier survival analysis stratified by risk score. **(G)** Multivariable Cox regression model tests for age, sex, TNM stage, polymerase ϵ (POLE) mutation status, and 4-gene mutation score. ***p < 0.001. **(H)** Mutation count per sample of gastric adenocarcinoma stratified by risk score. ****p < 0.0001 determined by Mann Whitney test.

The most common mutations of the 45 genes are missense substitutions. Other alterations include silent mutations, frameshift insertions and deletions, nonsense mutations, and other infrequent mutations. Both base substitution mutation and frameshift mutation can change the composition or sequence of amino acids in the polypeptide chain. According to the impact of different type mutations on DNA composition or sequence of amino acids, we scored these mutations, as follows: No mutation or Silent mutation: 0; Missense mutation, In-frame insertion or deletion: +1; Splice site mutation: +2; Nonsense mutation, frameshift insertion or deletion: +3; Multiple mutation: the maximum score of all (**Figure 3A**). There are four levels of mutations in total. Finally, we get the mutation score matrix (**Supplementary Table 3**). In order to select the most weighted genes, we used random survival forest algorithm (Ntree = 1,000, default parameters of Hemant Ishwaran algorithm) and set the 45 mutated genes score as variables in this model. We ranked these 45 genes by their importance from the random survival forest model (**Figure 3B**).

Since the mutation status is divided into four levels, we selected four genes to construct the model in order to preserve the effect of all mutation states on the prognosis and take into account the minimalist principle. Combining Kaplan–Meier survival analysis log-rank p-value, mutation coexistence pattern, and the result of random survival forest algorithm, we selected UTRN, MUC16, CCDC178, and HYDIN as candidates for an accurate prediction of survival in gastric adenocarcinoma patients. UTRN ranked first among the candidates in random survival forest model (**Figure 3B**). MUC16 had the highest mutation frequency among the 45 genes (**Table 1**). CCDC178 mutations seem to be an independent factor (**Figure 2A**). MUC16 and CCDC178 also ranked in the top 10 important candidates (**Figure 3B**). UTRN and CCDC178 mutations were not significantly co-occurring or exclusive with each other in their mutation pattern (**Figure 3C**). Based on the selection of the three genes, using a computer to build the multivariate Cox model repeatedly, HYDIN was selected among the genes that had no obvious co-occurring or exclusive mutations with UTRN and CCDC178.

Of the 437 patients in TCGA cohort, the four genes were altered in 177 patients (40.5%). Consistently, gastric adenocarcinoma samples with the four mutated genes had a significantly higher mutation load (**Figures 3D, E**; Mann–Whitney test, p < 0.0001).

The mutated genes chosen from the previous step were constructed into the multivariate Cox proportional model to calculate the coefficients in TCGA cohort, thereby establishing the risk formula by which a risk score for each patient was calculated. Risk score = −0.1445* (mutation score of MUC16) − 0.459* (mutation score of UTRN) - 0.332* (mutation score of HYDIN) + 0.3102* (mutation score of CCDC178). Cutting off by 0, we defined risk score <0 as low-risk group, risk score = 0 as medium-risk group, and risk score >0 as high-risk group. Patients in the low-risk group had a markedly longer overall survival than those in the medium-risk group, and high-risk group had the shortest overall survival (**Figure 3F**; p < 0.0001, by log rank). The correlation between the four-gene-based signature and overall survival remained statistically significant after controlling for age, sex, TNM stage, and POLE mutation status in the multivariate model [hazard ratio (HR), 1.88; 95% CI, 1.33–2.7; p < 0.001] (**Figure 3G**). A significantly higher mutation count was also observed in gastric adenocarcinoma samples within the low-risk group (**Figure 3H**; Mann–Whitney test p-value <0.0001). So, the signature of the four-gene mutation would*nbsp;be a good prediction for survival of gastric adenocarcinoma patients.

### Independent Validation of Four−Gene-Based Risk Score in the Tianjin Cohort

**Figure 4:**
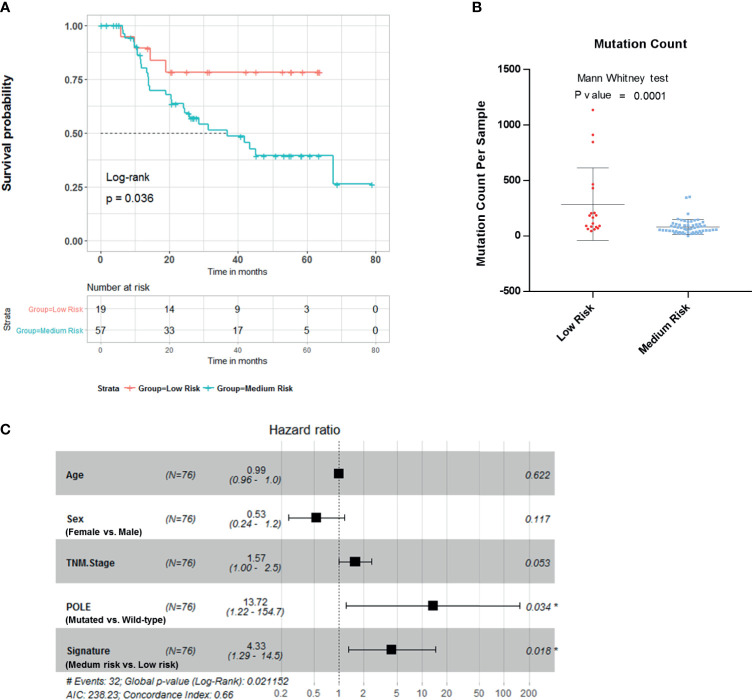
Validation of the risk score in Tianjin gastric adenocarcinoma cohort. **(A)** Kaplan–Meier survival analysis stratified by risk score. **(B)** Mutation count per sample of gastric adenocarcinoma stratified by risk score. **(C)** Multivariable Cox regression model tests for age, sex, TNM stage, polymerase ϵ (POLE) mutation status, and 4-gene mutation score. *p < 0.05.

To further evaluate the clinical value of this four-gene mutation signature, we used Tianjin cohort dataset with survival information. Kaplan–Meier survival analyses showed low-risk scores were significantly associated with better survival outcomes (**Figure 4A**; log-rank test, p = 0.036). Significantly higher mutation count was also observed in Tianjin cohort gastric adenocarcinoma samples in the low-risk group (**Figure 4B**; Mann–Whitney test p-value <0.0001). Multivariable Cox regression analysis also showed that the association of the risk score with overall survival was statistically significant after controlling for age, sex, TNM stage, and POLE mutation status (**Figure 4C**; HR, 4.33; 95% CI, 1.29–14.5; p = 0.018).

## Discussion

We analyzed 437 gastric adenocarcinoma samples from TCGA cohort and 78 gastric adenocarcinoma samples from a Tianjin cohort for survival prediction genes. We have identified and verified 45 mutated genes related to survival from 17,431 protein-coding genes in STAD. The GO enrichment showed that these genes play an important role in “cell–cell adhesion *via* plasma–membrane adhesion”, “extracellular matrix component”, and “alpha-catenin binding”. Obviously, these genes played a pivotal role in cancer metastasis. After that, we ranked the 45 survival−related mutated genes by random survival forest algorithm. Whole-genome sequencing is expensive, and data analysis takes a long time. The aim of this study is to develop a cheap and practical prognostic tool that can be accomplished by PCR. Combining Kaplan–Meier survival analysis log-rank p-value, mutation coexistence pattern, and the result of random survival forest algorithm, we selected four-gene mutation status to build a prognostic risk score that could be transformed into the clinical setting. Gastric adenocarcinoma samples classified into low-risk group had a significantly higher tumor mutation load and better survival outcome. The association between the four−gene-based risk score and overall survival was independent of mutations in POLE mutation status, age, sex, and TNM stage. Many studies have reported the association between these genes and tumors.

The deletion of chromosome 6q has been extensively mapped in a variety of tumors (11, 12). UTRN is located in this region, which encodes dystrophin. UTRN is a tumor suppressor inducing cell transformation when expressed in an antisense orientation. Studies showed decreased expression and inactivation mutations of UTRN in tumors. Expression of a wild-type UTRN in breast cancer cells inhibited tumor cell growth *in vitro* and reduced their tumor potential in nude mice (13). HYDIN is a gene whose impaired function has been linked to abnormal ciliary function, dyskinesia, and brain abnormalities (14, 15). HYDIN-derived sequences are targeted by the adaptive immunity in patients with cancer (16). Somatic mutations in HYDIN were found in breast cancer samples (17–19). MUC16, encoding a type I transmembrane mucin protein (20, 21), is frequently mutated in multiple types of human cancer (22). MUC16 was reported to modulate immune response to cancer (23–25). CA125 is a repeating peptide epitope of the mucin MUC16 (26, 27). MUC16 mutations were found to be associated with higher tumor mutation load, better survival outcomes, and immune response and cell cycle pathways in gastric adenocarcinoma (28). The CCDC178 is an 867-amino acid polypeptide and belongs to the superfamily of coiled-coil domain-containing protein. CCDC178 was reported to be mutated in hepatocellular carcinoma (29) and gastric carcinoma (30). CCDC178 associated with BRCA1-associated protein 2 (BRAP2), a negative regulator of extracellular signal-regulated kinase (ERK) pathway, and promoted its degradation (31). CCDC178 deficiency impaired the ERK activation in hepatocellular carcinoma (31). In our study, CCDC178 mutations were significantly associated with a poor survival outcome in gastric adenocarcinoma. The relative transcriptional level of CCDC178 was significantly downregulated in several types of carcinoma compared with adjacent non-cancerous tissues in TCGA cohorts (**Supplementary Figure 1**).

Compared with mutation detection, measurement of gene expression in cross-platform is unstable. Due to the lack of reproducibility and standardization, its clinical application may be limited. Recently, high-throughput sequencing technologies have been widely utilized in clinical cancer research. Compared to normal tissues, many high/low expressed proteins and mutated genes in tumor cells were identified. Combining several altered genes together may be feasible in predicting gastric adenocarcinoma risk and prognosis. In our study, the four genes (UTRN, MUC16, CCDC178, and HYDIN) were mutated in 177 patients (40.5%) in TCGA STAD cohort. The risk score is powerful and accurate in prognostic stratification. Our work provided an advanced method toward clinical applications of gene mutation profiling in STAD, especially in future personalized prediction and precision medicine.

However, our study has several limitations. The number of samples with follow-up data in the Tianjin cohort was limited. No CCDC178 mutation was detected in the Tianjin cohort. Gastric adenocarcinoma is a heterogeneous disease. Molecular subtyping can encompass this heterogeneity and provide useful clinical information. Prognostic tool constructed on the basis of anatomic site, histopathology, and molecular subtype may be more powerful and accurate. Considering the number of samples, anatomic site, histopathology, and molecular subtype were not included in this study. So, more prospective studies are necessary to further validate the reliability and stability of this risk score.

## Conclusions

A four-mutated gene risk formula was developed that correlated with the overall survival of patients with gastric adenocarcinoma using a multivariable Cox regression model. In two independent genomic datasets from TCGA and Tianjin cohorts, low risk scores were associated with higher tumor mutation loads and improved outcome in patients with gastric adenocarcinoma. This finding may have implications for prognostic prediction and therapeutic guidance for gastric adenocarcinoma.

## Data Availability Statement

Publicly available datasets were analyzed in this study. These data can be found here: https://portal.gdc.cancer.gov http://www.pnas.org/lookup/suppl/doi:10.1073/pnas.1422640112/-/DCSupplemental.

## Ethics Statement

Ethical review and approval was not required for the study on human participants in accordance with the local legislation and institutional requirements. Written informed consent for participation was not required for this study in accordance with the national legislation and the institutional requirements.

## Author Contributions

BZ: statistical analysis, analysis and interpretation of data, drafting of the manuscript. YL: critical revision of the manuscript for important intellectual content. LY: critical revision of the manuscript for important intellectual content. YC: critical revision of the manuscript for important intellectual content. All authors contributed to the article and approved the submitted version.

## Conflict of Interest

The authors declare that the research was conducted in the absence of any commercial or financial relationships that could be construed as a potential conflict of interest.

## Publisher’s Note

All claims expressed in this article are solely those of the authors and do not necessarily represent those of their affiliated organizations, or those of the publisher, the editors and the reviewers. Any product that may be evaluated in this article, or claim that may be made by its manufacturer, is not guaranteed or endorsed by the publisher.

## Supplementary Material

Supplementary Figure 1 CCDC178 is downregulated in several kinds of human carcinoma. **(A)** Lollipop plot displaying mutation distribution and protein domains for CCDC178 in STAD with the labeled recurrent hotspots. **(B)** Box plot displaying the gene expression profile across tumor samples and paired normal tissues. Each dot represents expression of samples. BLCA, Bladder Urothelial Carcinoma; BRCA, Breast invasive carcinoma; CESC, Cervical squamous cell carcinoma and endocervical adenocarcinoma; KICH, Kidney Chromophobe; KIRC, Kidney renal clear cell carcinoma; KIRP, Kidney renal papillary cell carcinoma; STAD, Stomach adenocarcinoma; UCEC, Uterine Corpus Endometrial Carcinoma.
