# Supplementary figures and images for: A Four−Gene-Based Risk Score With High Prognostic Value in Gastric Cancer
Source: Front Oncol. 2021 Sep 2;11:584213. doi: 10.3389/fonc.2021.584213 (PMC8443773; doi:10.3389/fonc.2021.584213)

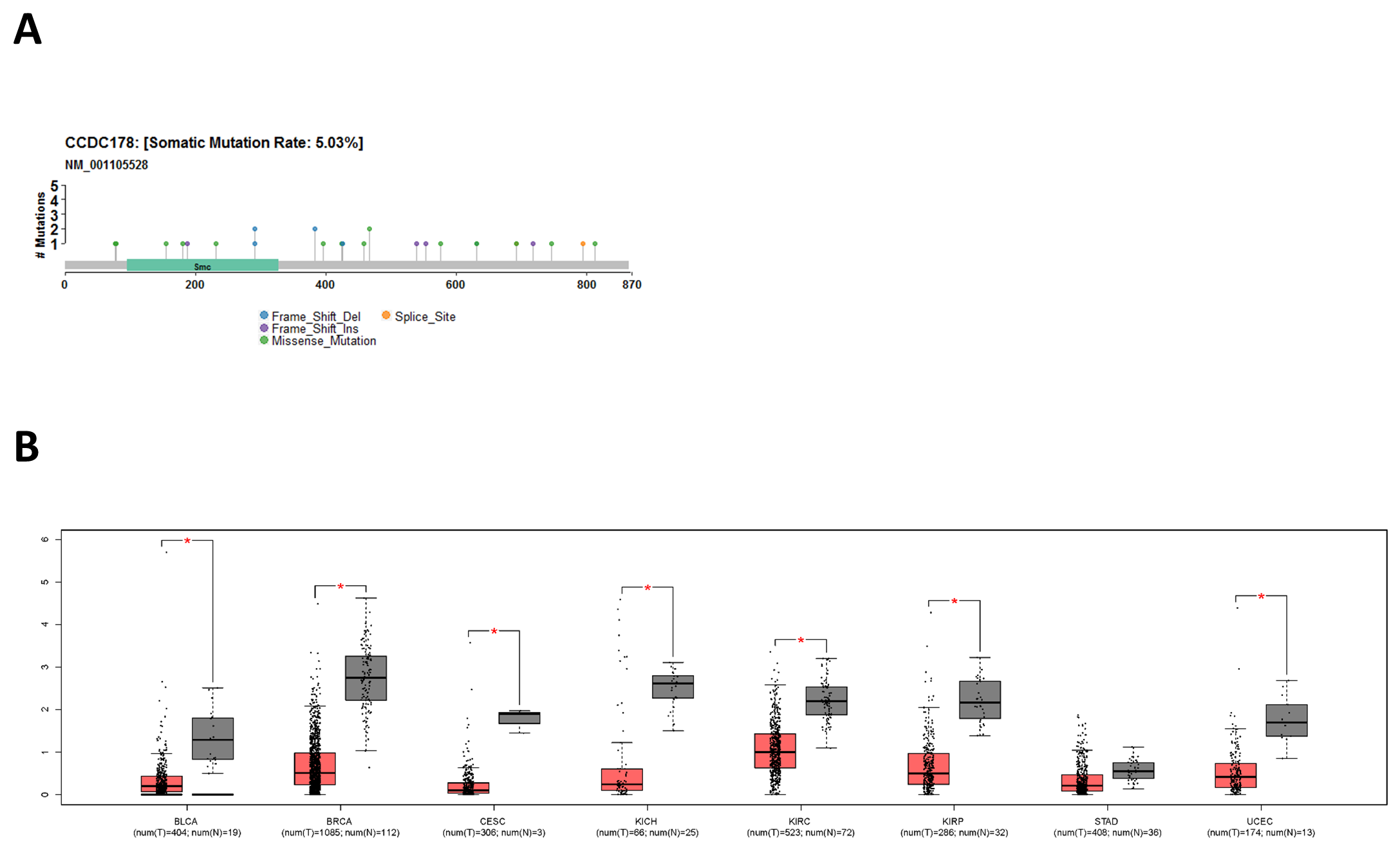

Supplement: Supplementary Figure 1 — CCDC178 is downregulated in several kinds of human carcinoma. (A) Lollipop plot displaying mutation distribution and protein domains for CCDC178 in STAD with the labeled recurrent hotspots. (B) Box plot displaying the gene expression profile across tumor samples and paired normal tissues. Each dot represents expression of samples. BLCA, Bladder Urothelial Carcinoma; BRCA, Breast invasive carcinoma; CESC, Cervical squamous cell carcinoma and endocervical adenocarcinoma; KICH, Kidney Chromophobe; KIRC, Kidney renal clear cell carcinoma; KIRP, Kidney renal papillary cell carcinoma; STAD, Stomach adenocarcinoma; UCEC, Uterine Corpus Endometrial Carcinoma. [file Image_1.tif]
